# Palladium(II) Metal Complex Fabricated Titanium Implant Mitigates Dual-Species Biofilms in Artificial Synovial Fluid

**DOI:** 10.3390/antibiotics12081296

**Published:** 2023-08-08

**Authors:** Sowndarya Jothipandiyan, Devarajan Suresh, Saravanan Sekaran, Nithyanand Paramasivam

**Affiliations:** 1 Biofilm Biology Laboratory, Centre for Research on Infectious Diseases (CRID), School of Chemical and Biotechnology, SASTRA Deemed University, Tirumalaisamudram, Thanjavur 613 401, Tamil Nadu, India; sowndarya@scbt.sastra.ac.in; 2 Department of Chemistry, School of Chemical and Biotechnology, SASTRA Deemed University, Thanjavur 613 401, Tamil Nadu, India; suresh_d@scbt.sastra.edu; 3 Department of Prosthodontics, Saveetha Dental College and Hospitals, Saveetha Institute for Medical and Technical Sciences, Chennai 600 077, Tamil Nadu, India

**Keywords:** artificial synovial fluid, biocompatibility, dual-species biofilms, implant, Palladium(II) metal complex

## Abstract

Metallodrugs have a potent application in various medical fields. In the current study, we used a novel Palladium(II) thiazolinyl picolinamide complex that was directly fabricated over the titanium implant to examine its potency in inhibiting dual-species biofilms and exopolysaccharides. Additionally, inhibition of mono- and dual-species biofilms by coated titanium plates in an in vitro joint microcosm was performed. The study was carried out for 7 days by cultivating mono- and dual-species biofilms on titanium plates placed in both growth media and artificial synovial fluid (ASF). By qPCR analysis, the interaction of co-cultured biofilms in ASF and the alteration in gene expression of co-cultured biofilms were studied. Remarkable alleviation of biofilm accumulation and EPS secretion was observed on the coated titanium plates. The effective impairment of biofilms and EPS matrix of biofilms on Pd(II)-**E**-coated titanium plates were visualized by Scanning Electron Microscopy. Moreover, coated titanium plates improved the adhesion of osteoblast cells, which is crucial for a bone biomaterial. The potential bioactivity of coated plates was also confirmed at the molecular level using qPCR analysis. The stability of coated plates in ASF for 7 days was examined with FESEM-EDAX analysis. Collectively, the present study provided an excellent anti-infective effect on Pd(II)-**E**-coated titanium plates without affecting their biocompatibility with bone cells.

## 1. Introduction

The use of orthopedic implants has become essential to augment or replace the function of bone in several defects, including injury and bone diseases in the elderly population [1]. Orthopedic implants consist of temporary fixatives and permanent prostheses. Temporary implants include plates, screws, and wires that are fixed to the broken bone until healing occurs. Permanent prostheses are used to replace the damaged bone and reside permanently inside the body [2]. 

Titanium (Ti) is the most widely used orthopedic implant material because of its strength, corrosion resistance, and excellent biocompatibility with the host tissue for bone formation [3,4]. Though a titanium implant favors host tissue attachment, it is also prone to microbial colonization and biofilm formation [4]. Moreover, there is always a competition between the host cell (Osteoblast) and contaminating bacteria to occupy the implant surface, which is termed the “race for the surface” [5]. If the host cells win the race, the biomaterial facilitates osseointegration and, at the same time, lessens bacterial infection. Conversely, if bacteria win the race, they colonize implants, form biofilms, and express virulence factors, which have a pivotal role in pathogenicity. Further, biofilms produce an extracellular polysaccharide matrix that acts as a physical barrier for antibiotics and host immune cells [6]. Thus, bacterial adhesion hinders the attachment of host cells to the surface, resulting in infection and implant failure [7]. 

Osteomyelitis is a serious microbial-induced bone infection that takes place during surgery, trauma-related injuries, and the implantation of biomaterials, resulting in damage to the bone and the surrounding tissue [6]. People with prostheses are susceptible to osteomyelitis, which is the most distressing of all biofilm infections and leads to deep bone infection [8,9]. Infections often cause prosthetic failure and require revision surgery, which leads to prolonged hospitalization with its associated costs [10]. Implant-associated osteomyelitis leads to chronic osteomyelitis even after the removal of the infected biomaterial. This is because bacteria from the biofilms get dispersed and colonize joint fluids [11,12]. Persistent implant infections caused by biofilms can ultimately lead to amputation and mortality [13]. 

The predominant biofilm-forming bacterial pathogens isolated from orthopedic implant-associated infections are *Staphylococcus aureus* and *Acinetobacter baumannii,* which are collectively grouped as ESKAPE pathogens [14,15]. *S. aureus* is a notorious pathogen responsible for chronic implant-associated osteomyelitis, which is found to be highly resistant to various drugs compared to *S. aureus* isolated from non-implant sites [15,16]. *A. baumannii* is a key Gram-negative pathogen known to cause orthopedic infections [17]. *A. baumannii* infections contribute to 30% of the mortality rate, and only a few therapeutic options exist [18]. It is also known as “Gram-negative MRSA” which has gained resistance against almost all antibiotics and has become a keystone pathogen in the clinical sector [19]. Infections arising through open fracture trauma and hardware implantation are frequently polymicrobial in nature, and the major fact observed in these infections is that virulence expression in polymicrobial biofilms is higher than in monospecies biofilms [20,21]. Recently, co-infections of Methicillin-resistant *Staphylococcus aureus* (MRSA) and *Acinetobacter baumannii* in open fracture injuries have been increasingly reported [22]. The serious concern of the co-infection of MRSA and *A. baumannii* polymicrobial communities in prosthetic joint infections imposes great difficulty in treatment as they are more resistant to antibiotics [23]. 

In order to alleviate implant infection, antibiotic-based titanium implants are being developed, but exposure of pathogens to antibiotics leads to drug resistance among them [3,4]. Therefore, coating anti-infectives on titanium without affecting bacterial growth is the ideal way to overcome antimicrobial resistance. However, in pursuit of controlling bacterial infection on the implant material, osseointegration, i.e., adherence and growth of bone cells, should not be affected or compromised. Hence, it is a prime requisite for any anti-infective compound to be coated on the biomaterial to have the ability to alleviate biofilms, support host cell attachment, and facilitate tissue integration [5,24]. To deal with antimicrobial resistance, we have synthesized novel thiazolinyl picolinamide complexes [25]. Our previous studies manifested that coating a Palladium(II) complex of N^∩^N^∩^N chelating thiazolinyl-picolinamide on a titanium implant effectively thwarted the virulence and biofilms of vital orthopedic implant infection-causing pathogens MRSA and *A. baumannii* [26,27]. Inspired by the above results, we were curious to know if the complexes could eradicate polymicrobial biofilms. Moreover, our previous study also demonstrated that Pd(II)-**E**-coated implants were non-toxic to osteoblastic cells as they exerted active metabolic activity, maintained cell integrity, and possessed cell spreading morphology [27]. Therefore, in the current study, Pd(II)-**E** was fabricated over titanium plates and explored against highly virulent dual-species biofilms of MRSA and *A. baumannii*. Moreover, in order to further investigate if the Pd(II)-**E**-coated titanium plates could render their anti-infective properties in a bone environment, a joint microcosm was created with artificial synovial fluid (ASF) with host proteins. In the present study, the whole experiment was carried out in artificial synovial fluid in order to mimic the native joint environment. Interestingly, effective inhibition of bacterial aggregation in ASF was noticed under Scanning Electron Microscopy (SEM) and fluorescent microscopic analysis even at day 7. A molecular gene expression study revealed downregulation of various virulence genes in biofilms developed on Pd(II)-**E**-coated titanium. The Pd(II)-**E**-coated titanium implant also enhanced osteoblast differentiation and bone mineralization, thereby expressing its biocompatibility. The overall outcome of the study reflected the development of biofunctionalized implants that suppress bacterial colonization while also encouraging bone integration.

## 2. Materials and Methods

### 2.1. Microbial Culture Condition and Metal Complex

Clinical isolates of Methicillin-resistant *Staphylococcus aureus* (GSA-44) and *Acinetobacter baumannii* AB2 (GenBank Accession No. MF443128) were used in this study. Bacterial strains were gifted by Dr. S. Karutha Pandian, Alagappa University, Karaikudi. Both strains were grown on soybean casein digest (SCD) agar (HiMedia) plates and incubated at 37 °C. Isolated colonies from the streaked plates were cultured in SCD broth and incubated at 37 °C under shaking conditions. Palladium(II)-based thiazolinyl picolinamide metal complex was synthesized and characterized by NMR [25]. The brief synthetic method for the complex is given here. A dichloromethane (5 mL) solution of 4-(Dimethylamino)pyridine (0.0063 mg, 0.052 mmol) was added into a well-stirred solution of Palladium precursor complex (0.030 g, 0.052 mmol) in acetonitrile (10 mL) at room temperature under nitrogen atmosphere. The reaction mixture was stirred for another 10 h. The bright yellow solution changed to pale yellow, and the solvent was removed under reduced pressure. The resultant yellow precipitate was washed with diethyl ether and vacuum-dried to afford Palladium(II) complex Pd(II)-E. Yield: 98% (0.031 g, 0.050 mmol). Melting point: >195 °C (decomposed). ^1^H NMR (300 MHz, DMSO-d_6_) δ 8.59 (d, *J* = 9.0 Hz, 2H, Ar-*H*) 8.36–8.27 (m, 1H, Ar-*H*), 8.23–8.18 (m, 2H, Ar-*H*), 8.14–8.02 (m, 1H, Ar-*H*), 8.04–7.96 (m, 1H, Ar-*H*), 7.87–7.74 (m, 2H, Ar-*H*), 7.55 (t, *J* = 9.0 Hz, 1H, Ar-*H*) 7.21–7.05 (m, 1H, Ar-*H*), 6.98 (d, *J* = 6.0 Hz, 1H, Ar-*H*), 4.49 (t, *J* = 9.0 Hz, 2H, N-C*H*_2_), 3.62 (t, *J* = 9.0 Hz, 2H, S-C*H*_2_), 3.16 (s, 6H, NMe_2_).


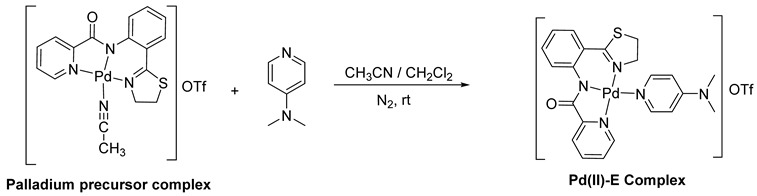


### 2.2. Evaluation of the Minimum Inhibitory Concentration (MIC) 

The minimum inhibitory concentration is the lowest concentration that inhibits the growth of bacteria. In our previous study, Pd(II)-**E** exhibited a MIC of 300 µg/mL against monospecies of Methicillin-resistant *Staphylococcus aureus* and *A. baumannii* [26,27]. Therefore, the same MIC concentration was used in the present study for MRSA and *A. baumannii* mono-species. As the complex Pd(II)-**E** was effective against both MRSA and *A. baumannii*, MIC was evaluated against dual-species MRSA and *A. baumannii* using different concentrations (100–1000 µg/mL). MIC was performed on 96-well flat-bottomed polystyrene microtiter plates (NEST Biotechnology, Korea). Initially, 100 µL of SCD broth was added in each well, and cultured broth from each strain (25 µL of MRSA and 25 µL *A. baumannii*) was added per well. Various concentrations of Pd(II)-**E** were added to their respective wells. DMSO at 0.1% was used as a vehicle control (VC) as it was used to dissolve the metal complex. Wells with media and culture alone served as controls. The plates were incubated at 37 °C, and MIC was evaluated from their optical density (OD) using a microtiter plate reader (Tulip, Lisaquant TS). MIC was further determined using the MTT assay [28]. 

### 2.3. Assessment of the Biofilm Inhibition Capacity of Pd(II)-**E** in Artificial Synovial Fluid

To check whether Pd(II)-**E** is effective in the joint environment, biofilm inhibition was evaluated by growing mono- and dual-species biofilms in ASF. Artificial synovial fluid (ASF) was prepared according to Pestrak et al., 2020, with slight modifications [29]. ASF preparation was as follows: 0.3% of Hyaluronic acid was dissolved in Ringer salt solution and autoclaved. To the solution, 19 mg/mL of Bovine serum albumin (BSA), 0.172 mg/mL of fibrinogen, and 450 µg/mL of fibronectin were added. All the components were dissolved in 1× PBS buffer and filter sterilized using a 0.22 µm syringe filter. 

In our previous study, the biofilm inhibitory concentration of monospecies of MRSA and *A. baumannii* in microbial culture media (soybean casein digest) was found to be 150 µg/mL [26,27]. Therefore, the same concentration was used in artificial synovial fluid to check whether Pd(II)-**E** is effective in ASF. Monospecies biofilms were developed by inoculating a suspension of 10^8^ cells/mL of MRSA and *A. baumannii* individually. Dual-species biofilms were developed in both microbial culture media and ASF. For dual-species biofilms, a 1:1 volume of bacterial suspension from each strain (10^8^ cells/mL from each strain) was used [30]. Biofilms were developed for 24 h with and without Pd(II)-**E**. Sub-MIC concentrations of Pd(II)-**E** at 150 µg/mL, 75 µg/mL, and 37.5 µg/mL were used for monospecies biofilms (MRSA and *A. baumannii*) according to Sowndarya et al., 2021; Sowndarya et al., 2022 [26,27]. For the dual-species biofilms, Pd(II)-**E** at 250 µg/mL, 125 µg/mL, and 62.5 µg/mL were screened as they are the sub-MIC (1/2, ¼, and 1/8) of the MIC (500 µg/mL). The biofilm studies were carried out using sub-MIC concentrations, as the focus of the study was to develop an alternate way to treat biofilm infections without killing bacteria.

### 2.4. Stability of Pd(II)-**E**-Coated Titanium Plates in Artificial Synovial Fluid

Grade 5 (Ti 6Al-4V) is the most commonly used titanium alloy in medical implants due to its high strength. Hence, Ti6Al-4V of size 1 × 1 cm was used throughout the study. Pd(II)-**E** at different concentrations (150 µg/mL for monospecies and 250 µg/mL for dual-species biofilms studies) were coated over the entire 1 × 1 cm titanium plates according to Sowndarya et al., 2021 [26]. Uncoated titanium plates served as controls. As the subsequent studies were carried out using Pd(II)-**E**-fabricated titanium plates, the stability of coated titanium plates was observed by immersing the plates in ASF at different time points (day 1 and day 7). The metal complex Pd(II)-**E** was dissolved in Tetrahydrofuran (THF), and the solution was loaded manually over titanium plates. The solvents used were allowed to evaporate, which caused the metal complex to deposit on the titanium. This forms a thin layer of coating on the titanium plate. The plates were then dried in a hot air oven and used for further studies [26]. The samples were gently washed with distilled water and dried. The surface characterization of the fabricated plates was performed using a Field Emission Scanning Electron Microscope (FESEM). In order to confirm the presence of all elements in the composition of Pd(II)-**E** adhered to a titanium plate after immersion, energy-dispersive X-ray spectroscopy (EDX) was performed. Elemental color mapping was performed to check for the distribution of elements of Pd(II)-**E** on titanium plates [27]. All the above analyses were carried out for day 1 and day 7 samples.

### 2.5. Biofilm Inhibitory Potency of Pd(II)-**E**-Coated Titanium Plates 

Mono- and dual-species biofilms were cultured on both the Pd(II)-**E**-coated and uncoated titanium plates. Pd(II)-**E** was fabricated over titanium plates by using the concentrations of 150 µg/mL for monospecies and 250 µg/mL for dual-species from the above experiment. The fabricated plates were transferred to 24-well polystyrene microtiter plates containing bacterial culture media (SCD broth) and ASF. Biofilm formation was evaluated on both Pd(II)-**E**-coated and uncoated Ti plates by crystal violet staining, as mentioned previously [31]. Mono- and dual-species biofilms were developed by incubating at 37 °C for 1 and 7 days, respectively. The comparison between the effectiveness of metal complexes between day 1 and day 7 was observed in SCD and ASF. Fresh media was supplemented every 24 h for the wells containing titanium plates. After the incubation period, the biofilms were stained with 0.4% crystal violet, and the dye bound to the biofilms was eluted in 70% ethanol. The eluted solution was then transferred to a 96-well microtiter plate, and absorbance was measured at 595 nm on a Microtiter plate reader (Tulip, Lisaquant TS). The percentage of biofilm inhibition was evaluated by the following formula:

$$\mathrm{Percentage}\;\mathrm{of}\;\mathrm{biofilm}\;\mathrm{inhibition}=\frac{\mathrm{OD}\;\mathrm{of}\;\mathrm{uncoated}\;\mathrm{Ti}\;\mathrm{plate}-\mathrm{OD}\;\mathrm{of}\;\mathrm{coated}\;\mathrm{Ti}\;\mathrm{plate}}{\mathrm{OD}\;\mathrm{of}\;\mathrm{uncoated}\;\mathrm{Ti}\;\mathrm{plate}}.$$

### 2.6. Evaluation of Viable Cells Residing in Biofilms 

As biofilms consist of bacterial cells and extracellular polymeric substances, quantification of biofilms by crystal violet staining does not differentiate between bacterial cells and the matrix. The viability of biofilm cells can be quantified based on their cultivability (CFU/mL) and metabolic activity (XTT assay). 

For the CFU agar plating method, biofilms were developed as mentioned previously for day 1 and day 7. Biofilms adhered to the titanium plates were dispersed or removed using 1× PBS. The biofilms in the PBS suspension were serially diluted and plated to enumerate the CFU/mL of biofilm viable cells for day 1 and day 7. The plates were incubated at 37 °C for 24 h [32].

The most common method to estimate the metabolically active cells of biofilm was the XTT (tetrazolium) reduction assay. To quantify the viable cells, titanium plates were placed in 24-well microtiter plates for growing biofilms. The titanium plates were gently washed using 1× PBS after incubation to remove the unbound cells. A total of 900 µL of the respective media and 100 µL of XTT were added to the biofilms adhered to the Ti plates. After incubating the plates in the dark for 5 h, metabolically active cells in the biofilms were measured at 490 nm [33].

From the earlier experiments, it was evident that ASF produced more biofilms than microbiological media. Therefore, further experiments, including microscopic studies and gene expression studies, were carried out by developing biofilms in ASF alone for both time periods. 

### 2.7. Live/Dead Fluorescent Staining of Biofilms

In order to visualize the coated plates with sub-MIC concentrations without affecting the bacterial cell membrane integrity, a fluorescence microscopy (Model-Eclipse Ts2, Serial No. 245859) study was performed. Biofilms were stained using the BacLight LIVE/DEAD bacterial Viability Kit (Invitrogen) to identify whether Pd(II)-**E**-coated titanium plates prevented biofilm formation without killing bacterial cells. The staining solution was prepared by adding 3 µL of SYTO^®^ 9 and 3 µL of propidium iodide (PI) to 1 mL of filter-sterilized water. The titanium plates containing biofilms were transferred to a fresh 24-well microtiter plate. SYTO^®^ 9 stains viable cells, represented by green fluorescence, whereas PI stains dead cells in fluorescent green. From the above-prepared mixture, 200 µL were added to both the coated and uncoated titanium plates containing biofilms. The plates were incubated at 37 °C in dark conditions for 15 min. The excess dye was removed by washing the titanium plates in filter-sterilized water, and images were captured at 40× magnification [34].

### 2.8. Scanning Electron Microscopy (SEM) Examination of Biofilms

To visualize the morphology of extracellular matrix-encased biofilms, SEM was performed. Bacterial biofilm coverage on Pd(II)-**E**-coated and uncoated titanium plates was visualized by SEM. After 1 and 7 days of exposure to titanium plates in ASF, the non-adherent bacterial cells were washed with sterile water. The plates were placed in a 24-well plate and soaked in 2.5% glutaraldehyde for 1 h. Following fixation, the samples were immersed in a gradient concentration of ethanol (50%, 60%, 70%, 80%, 90%, and 100%) for dehydration. The samples were dried and coated with gold for SEM (VEGA3 TESCAN) evaluation to examine the surface morphology of biofilms [35].

### 2.9. Assessment of Polysaccharides in the ECM Matrix in ASF 

The variation in EPS production of biofilms grown on SCD and ASF was quantified for both time points (day 1 and day 7). The total amount of biofilm polysaccharides on Pd(II)-**E**-coated and uncoated titanium plates was estimated according to Gowrishankar et al. (2015) [36]. Biofilms on the titanium plate were extracted using 0.5% NaCl, 5% phenol, and 0.2% hydrazine solutions. The suspension was incubated in dark conditions for 1 h. Following incubation, the suspension was centrifuged at 10,000 rpm for 10 min, and absorbance was read at 490 nm in a microtiter plate reader (Tulip, Lisaquant TS).

### 2.10. Comparison of Virulence Gene Expression between Mono- and Dual-Species Biofilms Formed in ASF 

When two different species interact, it may lead to an alteration in the gene expression profile. Therefore, the gene expression patterns of mono- and dual-species biofilms were compared by growing in ASF for days 1 and 7. Their virulence gene expression in ASF was investigated using real-time PCR analysis. Initially, the uncoated plates were placed in 6-well plates containing ASF. Biofilms adhered to the titanium plates alone were collected using 1× PBS. RNA from the biofilm biomass was extracted using the TRIzol reagent (Sigma-Aldrich, St. Louis, MO, USA). cDNA synthesis from RNA was performed as mentioned in a high-capacity reverse transcription kit 260 (Applied Biosystem TM, San Diego, CA, USA). Real-time PCR reactions were prepared using SYBR green qPCR master mix (GeneSure™ SYBR Green qPCR Master Mix (2×), ROX Solution). Primers used to quantitate gene expression are tabulated (Table S1). Real-time PCR data was normalized using 16srRNA as an internal housekeeping gene to validate variation in gene expression of virulence and biofilm-related genes. The fold change of the targeted gene expression was determined using 2^−ΔΔCT^ method [37]. 

### 2.11. Downregulation of Virulence Gene Expression in Mono- and Dual-Species Biofilms in Pd(II)-E Impregnated Titanium Plates

To acquire a detailed understanding of the anti-biofilm activity of Pd(II)-**E**-coated plates, qPCR analyses were performed. RNA was isolated, as mentioned previously, to investigate the fold change of targeted genes from biofilms of Pd(II)-**E**-coated and uncoated titanium plates. 

### 2.12. Indirect Cytotoxicity Assessment of Pd(II)-**E**-Coated Titanium Plates Using Osteoblastic Cells 

Human osteoblastic cells (MG-63) were obtained from the National Centre for Cell Sciences (NCCS), Pune, India. Cells were cultured in Dulbecco’s Modified Eagle Medium (DMEM) containing 10% Fetal bovine serum (FBS) and incubated at 37 °C with a 5% CO_2_ supply to form a confluent cell monolayer. After trypsinization, the cells were subcultured for further studies. An indirect MTT assay was carried out to evaluate the cytotoxicity of the substance leached from the coated implant. Cells of concentration 5 × 10^4^/cm^2^ were seeded in 24-well plates to examine the cytotoxicity of the Pd(II)-**E**-coated titanium plates. The plates were sterilized by exposing them to UV radiation and using 70% ethanol. Coated (150 µg/mL and 250 µg/mL) and uncoated plates were immersed in DMEM for 24 h at 37 °C. After immersion, the conditioned medium was collected and added to the wells containing cells, and the plate was incubated for 24 and 48 h. Cells without conditioning medium treatment were referred to as control and those with 0.1% Triton X-100 as positive control. At the end of the incubation period, the medium was completely removed, and the cells were washed twice with 1× PBS. In each well, 1 mL of MTT at a concentration of 5 mg/mL was loaded and incubated under dark conditions for 4 h. Following incubation, DMSO was used to dissolve the formazan crystals formed by the cells, and the absorbance was read at 570 nm [38].

### 2.13. Quantification of Calcium Deposition by Alizarin Red Staining 

Differentiating osteoblast cells deposit calcium due to mineralization. To quantify calcium deposition, Alizarin red staining was employed. MG-63 cells were seeded over coated and uncoated titanium plates placed in 24-well plates containing osteogenic medium (10 mM -glycerophosphate, 10 nM dexamethasone, and 50 g/mL ascorbic acid in DMEM). Cells were maintained at different time points (day 7 and day 14) to determine the mineralization. Fresh media was provided every three days. After incubation, the cells were washed with ice-cold 1× PBS, and the cells were subjected to 10% formalin for 1 h to attain fixation and treated with distilled water for 5 min for rehydration. Calcified deposits of the cell were stained with Alizarin red (1% Alizarin red in 2% ethanol, pH 4.2) for 10 min. The stained deposits were rinsed twice with deionized water to remove the unbound dye. It was then solubilized using 10% acetic acid, followed by incubation for 10 min at 80 °C. The neutralization of the solution was performed with ammonium hydroxide and quantified at 405 nm [39].

### 2.14. Real-Time PCR Analysis of Osteoblast Differentiation Marker Genes

To determine osteoblast differentiation, Pd(II)-**E**-coated and uncoated plates were placed in 6-well plates containing osteogenic medium. A quantity of 5 × 10^5^ MG-63 cells was seeded in each well and incubated at different time points (day 7 and day 14). Total RNA was isolated by the TRIzol method. cDNA was reverse transcribed from RNA according to the manufacturer’s instructions (Invitrogen, Carlsbad, CA, USA). RT-PCR was performed using SYBR reagent (Invitrogen). Glyceraldehyde 3-phosphate dehydrogenase (GADPH) was used as an internal control. The fold change in gene expression was calculated using the 2^−ΔΔCT^ method [39]. Primers used in the study were listed in Table S2.

### 2.15. Statistics

The data plotted in the graph represent the mean of the triplicates, and error bars indicate the standard deviation of the mean. The statistical significance between the control and treated groups was analyzed using GraphPad Prism. 

## 3. Results

### 3.1. Minimum Inhibitory Concentration of Pd(II)-**E** against Dual-Species Planktonic Cells 

The dual cultures of MRSA and *A. baumannii* were checked for their susceptibility to the Pd(II)-**E** metal complex. Pd(II)-**E** showed inhibition at a concentration of 500 µg/mL. The lowest concentration that inhibits the growth of bacteria was confirmed with the MTT assay. Viable cells convert MTT to a purple-colored formazan product. The cells that are killed will lose the ability to form a formazan product, thereby remaining colorless. The metabolically inactive dead cells were observed in the wells supplemented with Pd(II)-**E** ranging from 500 to 1000 µg/mL. Therefore, 500 µg/mL was determined as the MIC (Supplementary Figure S1).

### 3.2. Pd(II)-**E** Inhibited Mono- and Dual-Species Biofilms in ASF

Supplementation of Pd(II)-**E** actively suppressed the monospecies biofilms of MRSA and *A. baumannii* grown in ASF to a maximum of 75% and 73% at its sub-MIC (150 µg/mL), respectively (Supplementary Figure S2A). In addition, Pd(II)-**E** with 250 µg/mL (sub-MIC) had a significant reduction in the biomass of dual-species biofilms in the culture media as well as ASF. Pd(II)-**E** reduced biofilms of dual species in a dose-dependent manner. Maximum biofilm disruption was observed to be 69% and 73% in dual-biofilms grown in SCD and ASF supplemented with Pd(II)-**E** at its 1/2 MIC (250 µg/mL). Other sub-MIC of Pd(II)-**E** (125 µg/mL and 62.5 µg/mL) exhibited 6–52% inhibition of dual-species biofilms in both SCD and ASF, respectively (Supplementary Figure S2B). Overall, the results demonstrated that Pd(II)-**E** acts as an active biofilm-disrupting agent against both monospecies and dual-species biofilms of both MRSA and *A. baumannii*. The biofilm inhibitory concentration of Pd(II)-**E** against monospecies was 150 µg/mL, and for dual-species, it was 250 µg/mL, which was effective in both SCD and ASF.

### 3.3. Pd(II)-**E** Coating on Titanium Plates Remains Stable in ASF

**Figure 1:**
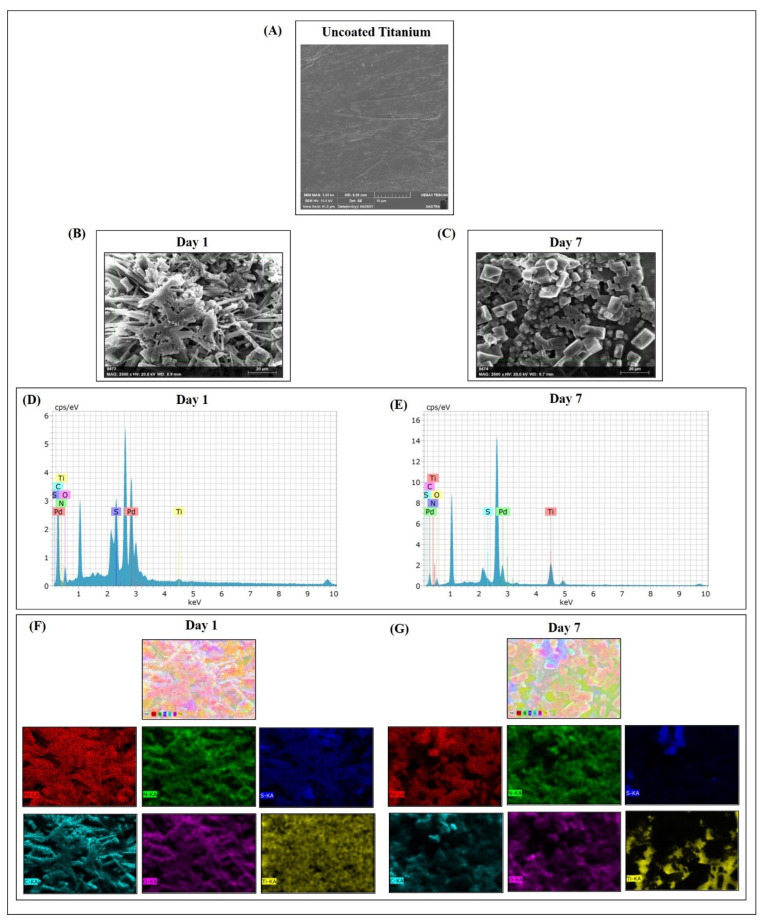
SEM image of an uncoated titanium plate (**A**). Representative FESEM images of Pd(II)-**E** coated titanium plates immersed in artificial synovial fluid on days 1 (**B**) and 7 (**C**). EDAX analysis of day 1 (**D**) and day 7 (**E**) coated titanium plates after incubation in ASF. Elemental color mapping of Pd(II)-**E**-coated titanium plates represents the elemental distribution of metal complex on days 1 (**F**) and 7 (**G**).

The long-term stability of Pd(II)-**E**-coated titanium plates was verified by placing the coated titanium plates in ASF for up to 7 days. After immersing coated plates in ASF, they were visualized in FESEM-EDAX. FESEM confirmed the uniform coating of Pd(II)-**E** where the metal complex was deposited over the titanium plate on both days 1 and 7 (Figure 1B,C). EDAX analysis revealed the elemental composition of the coated layer on the implant. The presence of various elements (Palladium, nitrogen, sulfur, carbon, oxygen, and titanium) in Pd(II)-**E** remains stable even after 7 days of incubation (Figure 1D,E). The detailed analysis of the elemental distribution of Pd(II)-**E** on the titanium plate was performed using elemental color mapping. Figure 1F,G of elemental color mapping visualized that all the elements of Pd(II)-**E** consisting of Palladium, carbon, sulfur, titanium, oxygen, and nitrogen, were properly distributed all over the titanium plate. The above results indicated that Pd(II)-**E**-fabricated titanium plates were stable in artificial synovial fluid for up to 7 days. As the fabricated metal complex is stable on the titanium surface, the anti-biofilm and biocompatible studies were performed using Pd(II)-**E**-coated titanium plates. 

### 3.4. Pd(II)-**E** Fabricated Titanium Plates Impede Biofilm Adherence

**Figure 2:**
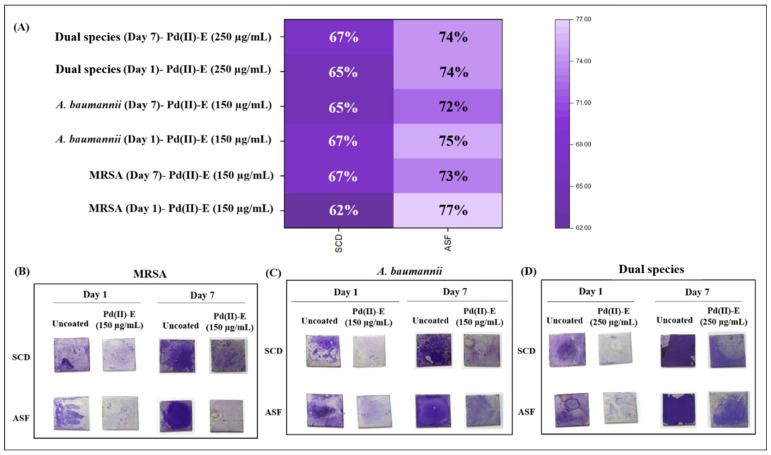
Biofilm inhibition of Pd(II)-**E**-coated titanium plates. (**A**) A heat map representing percentage biofilm inhibition of MRSA, *A. baumannii*, and dual species biofilms developed in SCD and ASF on day 1 and day 7. Representative titanium plates showing biofilms of MRSA (**B**), *A. baumannii* (**C**), and dual species biofilms (**D**) stained with crystal violet dye on days 1 and 7. SCD-soybean casein digest; ASF-Artificial synovial fluid.

We investigated the biofilm-forming ability of mono- and dual-species biofilms on Pd(II)-**E**-coated titanium plates immersed in SCD and ASF for up to 7 days. CV staining was performed to quantify the biomass adhered to the surface of the titanium plate. The intensity of the crystal violet stain on the uncoated plates was high. It was found that the maximum biofilm inhibition was observed in ASF compared to SCD for days 1 and 7 (Figure 2A–D). About 74–77% of biofilm inhibition was noticed for mono- and dual-species biofilms grown in ASF on day 1. Similarly, for day 7, the biomass of about 72–74% was reduced in ASF. In SCD, 62–67% biomass inhibition was observed in all the test strains (MRSA, *A. baumannii*, and dual-species (MRSA and *A. baumannii*)) for both days 1 and 7. 

### 3.5. Viable Biofilm Cells Were Lowered in Pd(II)-**E**-Coated Titanium Plates

As CV dye stains all the biomass, including extracellular matrix and live and dead cells, we estimated viable cells residing in biofilms adhered to implants by CFU/mL and XTT assays. Compared to uncoated plates, coated plates effectively decreased Log CFU/mL of biofilms (Supplementary Figure S3A,B). The fewer biofilm cells in Pd(II)-**E** coated titanium correlated with the XTT metabolic assay (Supplementary Figure S3C–F). The dehydrogenase enzyme from live cells of biofilm reduces XTT tetrazolium salt to form a purple formazan product. The biofilms developed on uncoated plates produce more formazan, depicting a purple color, due to the presence of more functional biofilm cells. Coated plates produced very little formazan even after 7 days of incubation, as only a few cells adhered to the coated plates (Supplementary Figure S3C–F).

**Figure 3:**
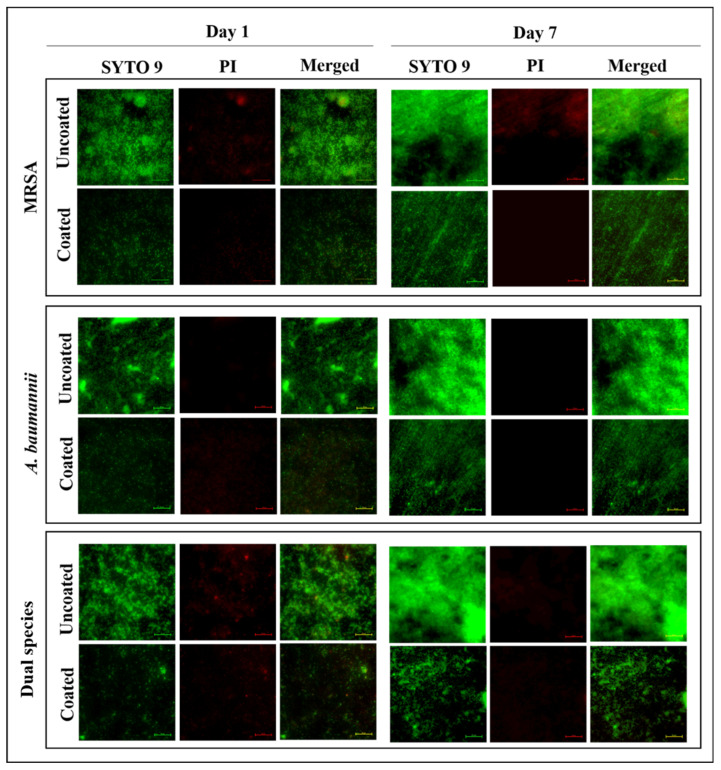
Fluorescent microscopic images showing live and dead cells of biofilms on titanium plates grown in artificial synovial fluid.

The results of the CFU/mL and XTT assays showed that the viability of biofilm cells was reduced on coated plates. To confirm that the reduction in viable biofilm cells on the coated plate is not due to anti-bacterial activity, Live/dead staining was performed. Fluorescence microscopic images aid in the clear visualization of live and dead cells in biofilms attached to the titanium plate. The results showed that on uncoated plates, bacterial agglomeration with a compact biofilm matrix was stained fluorescent green, as indicated by the high intensity of green fluorescence. On the other hand, the drastic onset of bacterial accumulation with significant morphological alteration in biofilms was observed on coated plates, as indicated by the low amount of green fluorescence. Cells from treated samples do not stain fluorescent red, indicating that coated plates do not damage bacterial cells (Figure 3). Thus, the coated titanium plate prevented biofilm formation without exhibiting antibacterial activity.

### 3.6. Coated Titanium Plates Disrupt the Extracellular Matrix of Biofilms in ASF

**Figure 4:**
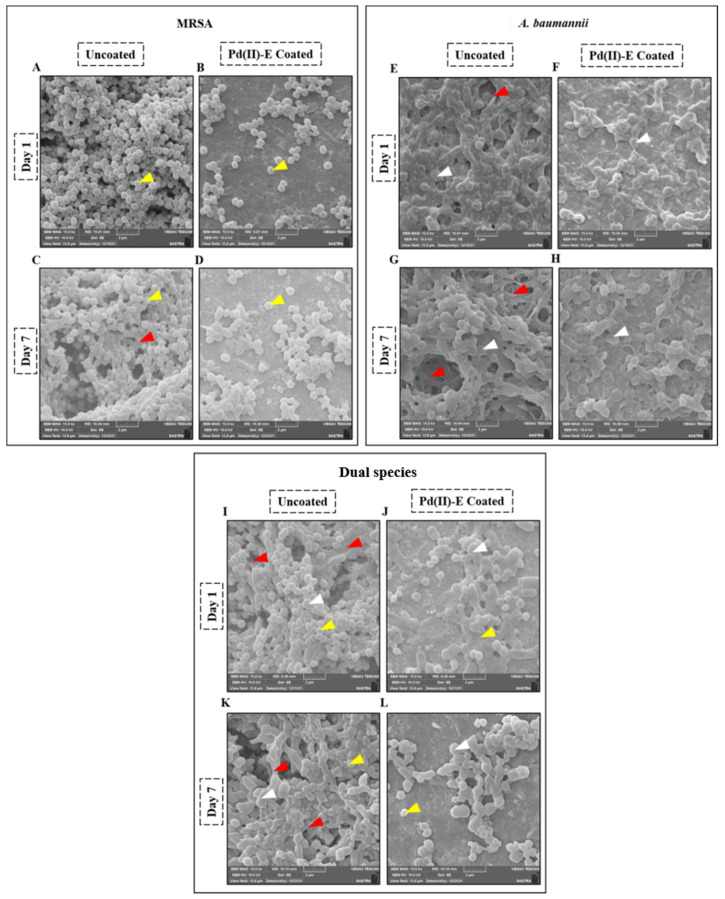
Scanning Electron Microscopic images of biofilms formed on coated and uncoated titanium plates in ASF. Micrograph representing biofilms of MRSA (**A**–**D**), *A. baumannii* (**E**–**H**), and dual species (**I**–**L**) on days 1 and 7. The yellow arrow indicates MRSA, the white arrow indicates *A. baumannii,* and the red arrow represents the EPS matrix of biofilms.

Scanning electron microscopic images provide the detailed structure of biofilms, including bacteria embedded in the extracellular matrix and cohesion attachment between the bacterial cells. The morphology and bacterial attachment of mono- and co-cultured biofilms grown in ASF over 7 days were visualized by SEM. Figure 4A shows MRSA on an uncoated titanium plate displaying a thick cluster of biofilms. It was clear that MRSA on coated titanium plates lacks the well-organized structure of biofilm (Figure 4B). From the visual analysis of Figure 4E, the uncoated samples seem to be covered with *A. baumannii* biofilms with complex matrix architecture. On the other hand, very few bacterial adhesions were noticed on treated titanium plates (Figure 4F). On co-culturing, thick aggregates of cocci and rods were observed, which appeared to be slimy (Figure 4I). Dual-species biofilms on coated plates displayed a discrete or small aggregate of bacteria (Figure 4J), indicating the effectiveness of Pd(II)-**E**-coated titanium plates against biofilms and EPS production of mono- and dual-species biofilm. 

A clear difference in the biofilm architecture of day 1 and day 7 images was observed as day 7 biofilms matured. Both mono- and co-cultured biofilms of day 7 on uncoated plates produced more densely structured biofilms with voluminous extracellular matrix (Figure 4C,G,K). Bacterial cells from biofilms on uncoated titanium were tightly packaged in multiple towers covered with a slimy layer. In contrast, the micrographs in Figure 4D,H,L appeared to have reduced bacterial attachment with less biofilm matrix material on Pd(II)-**E**-coated titanium plates. Thus, coated plates showed active prevention of biofilm adherence for about 7 days.

**Figure 5:**
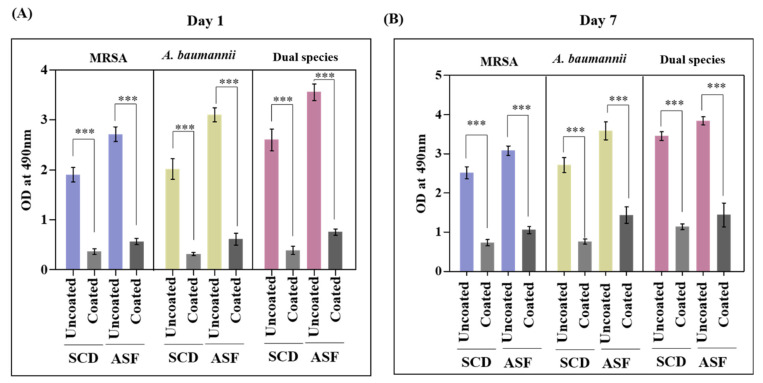
EPS quantification of biofilms from days 1 (**A**) and 7 (**B**). Graphs were plotted with means ± standard deviations of triplicates. Asterisks indicate statistical differences between the control and treated groups: *p* < 0.001 (***).

The results also corroborated the EPS quantification assay. A higher volume of Exopolysaccharides (EPS) was formed in ASF than in SCD, representing the components of ASF that enhance EPS production. Co-cultured biofilms produced rich EPS when compared to monospecies biofilms (Figure 5A). On quantifying the matrix EPS production of biofilms, as expected, EPS development was high on day 7 uncoated titanium plates (Figure 5B). Pd(II)-**E**-coated titanium plates effectively lessened EPS production in both SCD and ASF, even on the 7th day of incubation (Figure 5A,B). 

### 3.7. ASF-Induced Virulence Gene Expression by Pathogens 

**Figure 6:**
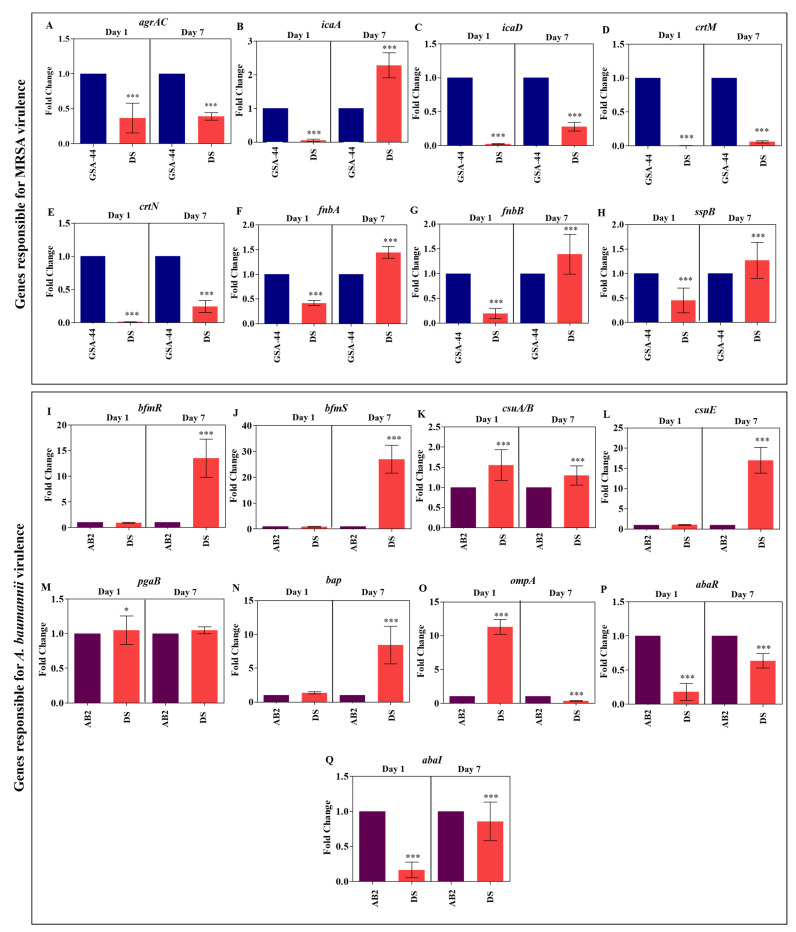
Comparison of virulence gene expression between mono- and dual-species (DS) biofilms formed on uncoated titanium plates at different time points (days 1 and 7) cultivated in artificial synovial fluid. Fold change difference in MRSA virulence gene expression between mono- and dual-species biofilms (**A**–**H**). Fold change in gene expression of *A. baumannii* virulence gene between mono- and dual-species biofilms (**I**–**Q**). Graphs were plotted with means ± standard deviations of triplicates. Asterisks indicate statistical differences between the control and treated groups: *p* < 0.001 (***), and *p* < 0.05 (*).

Biomaterials were in direct contact with body fluids. To understand how the ASF influences the virulence and biofilms of mono- and dual-species, their gene expression patterns were compared by relative fold change. Surprisingly, it was noticed that all the virulence genes of MRSA in dual-species biofilms were downregulated when compared to monospecies MRSA biofilms on day 1. On day 7, upregulation in several genes was noticed. A gene encoding polysaccharide intercellular adhesion (*icaA*) (Figure 6B and fibronectin binding protein, which promotes attachment to host tissue (*fnbA* and *fnbB*) (Figure 6F,G), was observed. Staphopain B (sspB), a cysteine protease that encourages the survival of MRSA in serum [40], was also elevated on day 7 of dual-species biofilms (Figure 6H). For *A. baumannii* genes, qPCR analysis revealed that genes responsible for surface attachment (*csuA/B*) (Figure 6K) and biofilm formation (*bap*) (Figure 6N) were upregulated on day 1 when *A. baumannii* was co-cultured with MRSA. A constant increase in gene expression of *bap* was observed until day 7 (Figure 6N). On day 7, *A. baumannii* genes responsible for pili-mediated surface attachment, including *bfmR* (12.52-fold), *bfmS* (25.98-fold), and *csuE* (15.99-fold), increased in dual-species biofilms (Figure 6I,J,L). *ompA*, a vital gene of *A. baumannii*, facilitates attachment to host fibronectin, which helps in aggregation and biofilm formation. Interestingly, *ompA* expression was increased to 10.29-fold in day 1 dual-species biofilms when compared to *A. baumannii* monospecies biofilms, but its expression was reduced to 0.35-fold in day 7 biofilms (Figure 6O). All the QS genes of both MRSA and *A. baumannii* (*agrAC, abaR*, and *abaI)* were downregulated in dual-species biofilms on both days 1 and 7 when compared to monospecies biofilms (Figure 6A,P,Q). From the result, it was envisaged that host protein binding factors are more crucial for co-cultured biofilms than quorum-sensing signaling systems. Therefore, interaction between two different species should be considered in the choice of therapy.

### 3.8. Coated Titanium Plates Downregulate Virulence Gene

**Figure 7:**
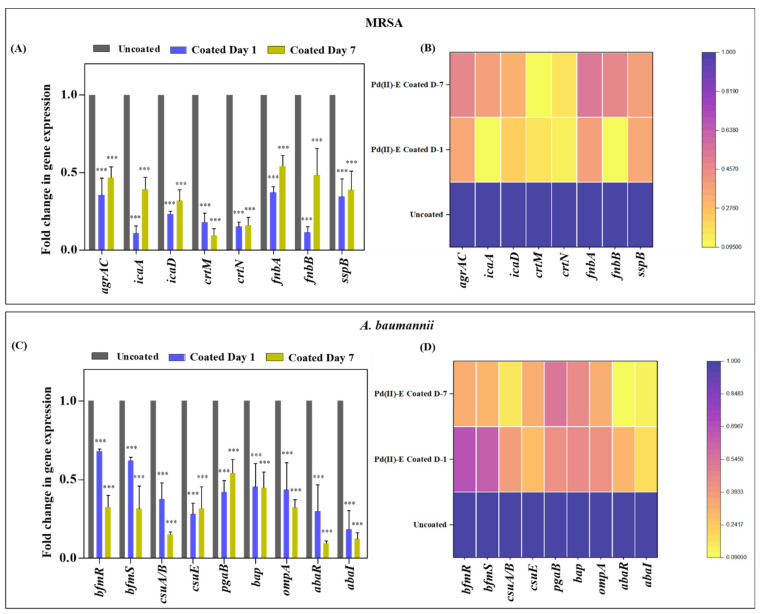
Real-time PCR analysis of monospecies biofilms. (**A**) Reduction in gene expression of MRSA biofilm on day 1 and day 7 titanium plates. (**B**) Heat map indicating fold change reduction of virulence gene expression of MRSA. (**C**) Fold change reduction of *A. baumannii* virulence genes on day 1 and day 7 titanium plates. (**D**) Heat map indicating fold change reduction of *A. baumannii* virulence gene expression on day 1 and day 7 biofilms. Purple indicates a high level of expression, and yellow indicates a low level of expression in the heat map. Graphs were plotted with means ± standard deviations of triplicates. Asterisks indicate statistical differences between the control and treated groups: *p* < 0.001 (***).

To get a clear insight into how Pd(II)-**E**-coated titanium plates impaired biofilms and the virulence of MRSA, qPCR was performed. MRSA biofilms are promoted by the expression of various factors. The master regulatory system AgrAC encodes the quorum sensing system of MRSA, which regulates several virulence factors of MRSA. The autoinducer peptide is the QS signaling molecule that was released outside the cell with the help of the cysteine protease SspB [41]. The expression of genes (*agrAC*, *sspB*) that enable QS was downregulated. Extracellular polysaccharide adhesion is mediated by the cooperation of the *icaA* and *icaD* genes. Thus, the extracellular polymeric matrix reduction might be due to the suppression of the *icaA* and *icaD* genes. MRSA causes infection by binding to host tissue, which is facilitated by fibronectin-binding proteins (*fnbA* and *fnbB*), and the expression of *fnbA* (0.63-fold on day 1 and 0.46-fold on day 7) and *fnbB* (0.89-fold on day 1 and 0.52-fold on day 7) was downregulated in coated titanium plates (Figure 7A,B). 

The gene expression profile of *A. baumannii* biofilms on coated titanium plates cultured in ASF was analyzed. BfmRS attributes biofilm formation to the abiotic surface. Therefore, controlling the gene expression of *bfmR* and *bfmS* subsequently decreased the expression of *csuA/B* and *csuE*, thereby reducing initial attachment and biofilm formation. The biofilm-associated surface protein encoded by *bap* facilitates intercellular aggregation of bacteria, where the expression of *bap* was reduced in the coated plate. Poly-β-1,6-*N*-acetylglucosamine (PNAG), encoded by *pgaB*, provides the structural integrity of the biofilm matrix that was downregulated in coated plates. Interaction of *A. baumannii* with host cells begins with the fibronectin-binding protein OmpA encoded by *ompA.* Attenuating the expression of *ompA* effectively prevents adhesion to host cells, thereby thwarting subsequent biofilm infection. A reduction in gene expression of *ompA* (0.57-fold on day 1 and 0.68-fold on day 7) was detected. AbaI and AbaR QS regulate the biofilm and surface motility of *A. baumannii*, which were also controlled by coated plates (Figure 7C,D).

**Figure 8:**
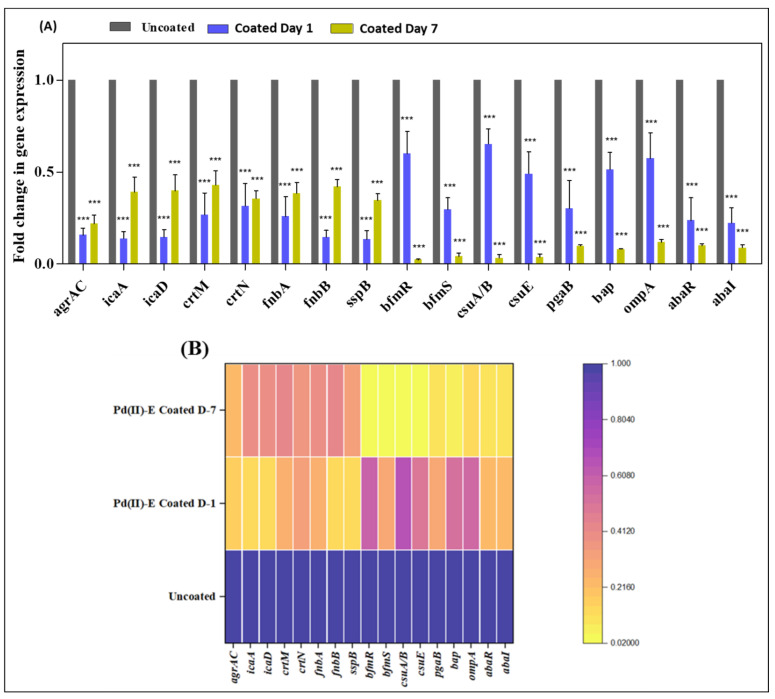
Real-time PCR analysis of dual-species biofilms. (**A**) Gene expression of dual-species biofilms on titanium plates cultivated in ASF. (**B**) Heat map representing dual-species biofilm gene expression. Purple indicates a high level of expression, and yellow indicates a low level of expression in the heat map. Graphs were plotted with means ± standard deviations of triplicates. Asterisks indicate statistical differences between the control and treated groups: *p* < 0.001 (***).

The outcome of qPCR analysis of co-cultured biofilms revealed a decrease in *agrAC*, *icaA*, *icaD*, *crtM*, *crtN*, *fnbA* (0.74-fold on day 1 and 0.62-fold on day 7), *fnbB* (0.86-fold on day 1 and 0.58-fold on day 7), *sspB*, *bfmR*, *bfmS*, *csuA/B*, *csuE*, *pgaB*, *bap*, *ompA* (0.43-fold on day 1 and 0.88-fold on day 2), *abaR*, and *abaI* when Pd(II)-**E**-coated implants were placed in ASF (Figure 8A,B). The coated plates attenuate the above-mentioned virulence of both mono- and co-cultured biofilms up to day 7 in artificial synovial fluid.

### 3.9. Pd(II)-**E**-Coated Titanium Plate Is Biocompatible with Human Osteoblasts

**Figure 9:**
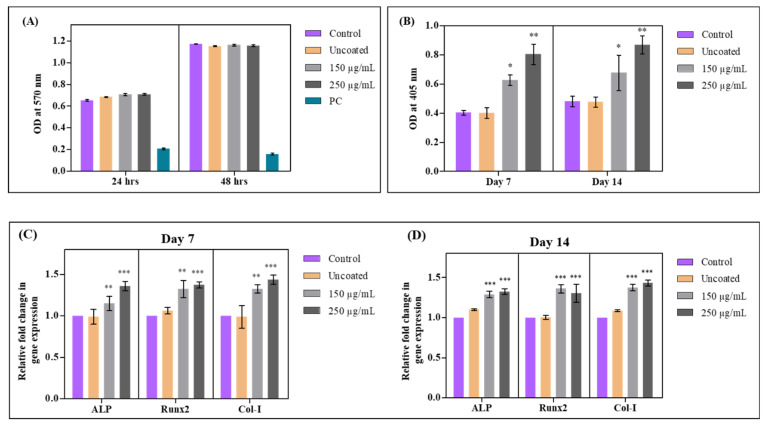
(**A**) Indirect MTT cytotoxicity assay of coated and uncoated titanium plates. (**B**) Quantification of biomineralization on coated and uncoated titanium plates at different time intervals, day 7. Real-time PCR analysis of genes involved in osteoblast differentiation on day 7 (**C**) and day 14 (**D**). Graphs were plotted with means ± standard deviations of triplicates. Asterisks indicate statistical differences between the control and treated groups: *p* < 0.001 (***), *p* < 0.01 (**), and *p* < 0.05 (*).

The in vivo implantation of implants immediately contacts tissue fluids and interacts with their cellular partners. Implant material intended for in vivo application should not exert any discrete toxicity on the cells. Metal ions are actively leached and released into the vicinity of tissues, which corresponds to biological action and toxicity. Therefore, we performed an indirect toxicity assessment using the standard procedure (ISO 10993-5:2009). The metabolic activity of the cells was quantified through an MTT assay, and the results depict the non-toxic nature of the coated implants (Figure 9A). There were no significant changes in metabolic activity among the treated and control groups. This shows that the coating of Pd(II)-**E** on titanium implants has no toxic effects on osteoblasts, proving it to be biocompatible.

### 3.10. Pd(II)-**E**-Coated Plates Enhanced Calcium Deposition

Next, we were interested in assessing the role of Pd(II)-**E** on osteoblast mineralization. We speculated that coated titanium plates may exhibit an enhancement in matrix mineralization. Therefore, we grew osteoblasts on the coated and uncoated plates under osteogenic conditions for a period of 7 and 14 days and assessed the extent of matrix mineralization by Alizarin red staining quantification. Pd(II)-**E** (250 µg/mL)-coated plates showed an increase in calcium deposition when compared with uncoated titanium plates and controls (Figure 9B). This indicates that Pd(II)-**E** coatings possess osteogenic properties, and a dose-dependent increase in matrix mineralization was observed.

### 3.11. Pd(II)-**E**-Coated Plates Promote Osteogenic Differentiation

Since we observed an increase in calcium deposition by osteoblasts upon their growth on coated titanium plates, we next assessed the molecular mechanism responsible for this effect. Therefore, we examined the mRNA expression levels of osteoblast differentiation markers by real-time RT-PCR analysis. Cells were grown on the coated and uncoated plates for a period of 7 and 14 days, followed by real-time PCR analysis to assess the expression levels of Runx2, ALP, and type 1 collagen (Col-I). The results revealed an elevated level in the expression of Runx2, ALP, and Col-I on Pd(II)-**E**-coated plates compared to other groups. A similar trend was observed at both the time points of 7 and 14 days. It is also interesting to note that there were no significant differences observed among Pd(II)-**E** (150 µg/mL) and Pd(II)-**E** (250 µg/mL) (Figure 9C,D).

## 4. Discussion

The synovial fluid protects the articular cartilage surface by reducing friction during movement and providing nutrition to the joint [42]. The rich proteinaceous nature of SF makes it an ideal substrate for the growth and aggregation of pathogens, promoting bacterial colonization of implants, which leads to PJI [43,44,45]. The clumping of bacteria in SF makes antibiotic treatment ineffective even at higher concentrations. Previously, our studies reported that Pd(II)-**E**-coated titanium implants reduced biofilm colonization and inhibited virulence factors of monospecies biofilms of MRSA and *A. baumannii* in microbial culture media (soybean casein digest) [26,27]. In keeping with our interest in whether Pd(II)-**E**-coated titanium retains its anti-infective efficacy in synovial fluid, the present finding deals with growing biofilm in artificial synovial fluid. The choice of using artificial synovial fluid is an alternative to collecting a large volume of synovial fluid from the patient. To our knowledge, the study delivers the first validation of co-cultured biofilms developed on titanium placed in artificial synovial fluid.

Implant-associated infections caused by bacterial biofilms are difficult to eradicate as they are intrinsically non-susceptible to antibiotic treatment. Dual-species biofilms, due to interspecies interaction, are more resistant to antibiotics, and eliminating these kinds of biofilms is a major task [46]. Therefore, developing infection-controlling implants that prevent bacterial adherence and simultaneously promote bone cell adherence on the implant is the need of the hour [47]. From the findings of the current study, it was clear that the Palladium thiazolinyl picolinamide metal complex was able to thwart highly virulent dual-species biofilms. In addition to that, it was found that Pd(II)-**E**-coated implants have good biocompatibility with bone cells without showing any toxic effects [26,27]. 

Host cell integration with the implant is achieved by the 7th day after implantation. Therefore, preventing bacterial colonization for up to 7 days would favor and allow the host cell to win the “run for the surface” and make the implantation successful [29,48]. Therefore, cell culture studies were also conducted using Pd(II)-**E**-coated titanium plates to check for their biocompatibility. All the studies were performed up to day 7 in ASF to check whether the coated titanium plates prevent mono- and dual-species biofilm formation. Mitigating biofilm formation for up to 7 days helps host cells attach to the implant surface, thereby winning the race for the surface. In uncoated samples, the bacteria, due to the presence of supplemented host proteins in the media, harbored almost the entire titanium surface. Microbial cells strongly encounter titanium plates due to the presence of ASF components and dense biofilm agglomeration with an extracellular matrix protecting the multiple towers of a bacterial population.

In the current study, to avoid anti-microbial resistance, sub-MIC concentrations of Pd(II)-**E** were chosen. This concentration does not affect or kill the planktonic cells, thereby preventing resistance against the drug. The sub-MIC (1/2 MIC) mitigated biofilm formation by about 73–75% on mono- and dual-species biofilms (Supplementary Figure S2). As Pd(II)-**E** possesses biofilm inhibition activity, it was coated over the implant, and its stability was inspected using FESEM EDAX analysis. Examining the coated plates after 7 days of immersion in artificial synovial fluid revealed that all the elements of the Pd(II)-**E** complex remain in the titanium plate. FESEM images showed uniform distribution of the Pd(II)-**E** complex coated over the titanium plates (Figure 1A,B). Every typical peak for each component of the complex found in the EDAX analysis of the day 1 sample was also found on day 7, indicating the stability of the complex coated over the plate (Figure 1C,D). Elemental color mapping showed the presence of all the elements of the Palladium complex in both day 1 and day 7 samples immersed in ASF (Figure 1E,F), which confirms the stability of Pd(II)-**E**-coated on titanium.

As the Pd(II)-**E** complex was stable in ASF even after its immersion for 7 days, biofilm studies were carried out using the coated plates. In this study, the Pd(II)-**E** complex adhered to the titanium implant was investigated for its anti-infective properties against MRSA and *A. baumannii* individually and in their co-culture biofilms for up to 7 days in ASF. These two strains are highly resistant to antibiotics, and their coexistence limits treatment options. The effectiveness of the coated implants was examined in different media by placing them in SCD and ASF. ASF consists of various protein molecules, including fibrinogen and fibronectin, which tend to form a conditioning film on the implant after implantation. These layers of host protein serve as a ligand for several receptors of the free-floating bacteria, promoting their attachment to the biomaterial, which aids in bacterial cohesion. Most of the biofilms retrieved from PJI are enriched with these proteins [49]. Fibrinogen and albumin in synovial fluid are the main components of bacterial aggregation [43]. MRSA interacts with fibrinogen and uses fibrinogen as a protective shield to evade the host’s immune response [50]. Hyaluronic acid, a proteoglycan, maintains the viscosity of the synovial fluid, favoring bacterial clustering and antibiotic tolerance [43]. Fibronectin-binding proteins of MRSA and *A. baumannii* interact with fibronectin present in biomaterial, which accumulates bacterial cells and develops biofilm infection [51,52]. Surprisingly, the inhibition of biofilms developed in ASF was higher in coated samples when compared to biofilms in SCD. Pd(II)-**E**-coated plates after 24 h caused about a 77% reduction in MRSA, a 75% reduction in *A. baumannii*, and dual-species biofilms in ASF, whereas a 62–67% reduction was observed in biofilms cultivated in SCD (Figure 2A). Even after 7 days of incubation, active biofilm inhibition was noticed on coated plates. In ASF, Pd(II)-**E**-coated titanium caused a 73% and 72% reduction of MRSA and *A. baumannii* biofilms, respectively, and a 74% reduction in dual-species biofilms. In SCD, coated plates lessened biofilms by about 65–67%, respectively (Figure 2A). The CV staining of biomass revealed that Pd(II)-**E**-fabricated titanium significantly reduced biofilms even in the presence of host-supplemented proteins. A previous study with a hexadentated macrocyclic complex of copper (II) derived from thiosemicarbazide showed potent antibiofilm activity against *S. aureus* for up to 72 h. The antibiofilm activity was due to its antimicrobial property against planktonic and biofilm cells [53]. Earlier studies reported a phendione-based copper and silver complex as an anti-infective agent against *Pseudomonas aeruginosa,* and they envisaged that its application on a medical device would thwart biofilm infections [54]. In the current study, the Palladium complex was coated in a titanium plate and tested against biofilms grown in ASF, analyzing its activity at different time points (day 1 and day 7). Biofilms are mostly presented in the form of dual-species communities on the surface, and their behavior would be changed due to their interaction with different species [30]. To minimize the dual-species biofilms adhered to the implant, the use of Pd(II)-**E**-coated titanium plates was explored against co-cultured biofilms grown in ASF. The present finding reported that Pd(II)-**E**-fabricated titanium plates inhibited dual-species biofilms without affecting the growth of planktonic cells. 

Protein fouling is a major issue in biomedical implant infections as it causes biofilm aggregation [55]. The scaffold of biofilm matrices contains host proteins and glycoproteins, which support bacterial adherence and nutrition [56]. In addition, *S. aureus* is capable of producing the coagulase enzyme, which activates prothrombin and converts fibrinogen to form a fibrin network. These fibrous complexes encircle bacteria and structure a biofilm matrix that masks pathogen recognition by immune cells [57]. It was noticed that EPS secretion was higher in biofilms grown on ASF than in SCD. The excess production of EPS may be due to the presence of host proteins in ASF. While co-culturing MRSA and *A. baumannii* in ASF, robust production of biofilms with more proteinaceous extracellular matrix secretion was observed when compared to monospecies (Figure 4I,K). The present study actively suppressed the matrix material (Figure 5) of biofilms, where many antibiotics fail. The reduction in EPS was due to the downregulation of various genes that are responsible for the interaction of bacterial cells with host proteins. FnbA and FnbB are the fibronectin-binding proteins of MRSA that help the bacteria interact with fibronectin to form bacterial aggregation and biofilm infection [23]. Likewise, OmpA, which is an outer membrane protein of *A. baumannii*, binds to fibrinogen and aids in initial attachment and associating biofilms [58]. In addition, Polysaccharide intercellular adhesion (PIA) or poly-β(1-6)-N-acetylglucosamine (PNAG) is one of the important components of the dual-species biofilm matrix, favoring the adhesion of pathogens on titanium surfaces [30]. As PNAG is found in both MRSA and *A. baumannii*, voluminous EPS is secreted by dual-species biofilms, promoting cell adhesion in interspecies biofilms. Moreover, PNAG/PIA is known to facilitate host colonization and antimicrobial resistance and reduce neutrophil responses [58]. All the targeted genes responsible for biofilm formation and extracellular matrix formation were downregulated in coated titanium plates (Figure 7 and Figure 8), thereby preventing bacterial contact with host proteins on biomaterials to prevent initial attachment and further accumulation of biofilms. 

The difference in phenotypic biofilm architecture between monospecies and dual-species biofilms and the effectiveness of Pd(II)-**E**-coated titanium against biofilms were visually compared by SEM and fluorescent microscopy. Day 7 biofilms showed intense biofilm formation with complex extracellular networks due to the presence of host proteins. Interestingly, the present study exhibited that *A. baumannii* produced excess extracellular matrix compared to MRSA on both days 1 and 7 (Figure 4E,G). In MRSA, biofilms developed dense bacterial aggregation on uncoated titanium implants, which may be due to their interaction with fibrinogen and fibronectin present in ASF (Figure 4A,C). On the other hand, Pd(II)-**E**-coated titanium plates prevented bacterial aggregation on day 1, and very little biofilm was seen on day 7 coated titanium plates (Figure 4B,D). This might be attributed to the fact that Pd(II)-**E**-coated titanium prohibited the interaction between host protein conditioning films and biomaterials, which mitigated bacterial attachment and lessened matrix production (Figure 4F,H).

**Figure 10:**
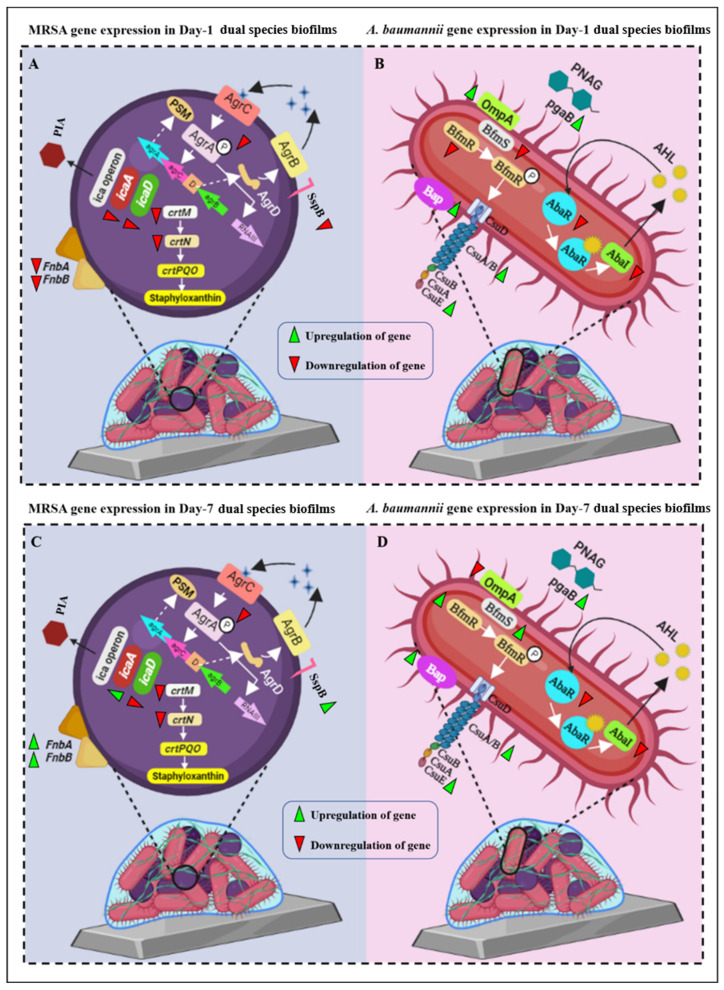
Graphical representation of virulence gene expression due to interaction of MRSA and *A. baumannii* in dual-species biofilms on an uncoated plate grown in artificial synovial fluid. (**A**) MRSA gene expression in day 1 dual-species biofilms, (**B**) *A. baumannii* gene expression in day-1 dual-species biofilms, (**C**) MRSA gene expression in day 7 dual-species biofilms and (**D**) *A. baumannii* gene expression in day-7 dual-species biofilms.

From the real-time PCR studies, it was speculated that host protein binding factors play a greater role in dual-species biofilm formation than quorum sensing signaling systems. In day 1 dual-species biofilms, it was noticed that *A. baumannii* was more predominant than MRSA (Figure 4I,K). A study has reported that *A. baumannii* has strain-specific anti-*S. aureus* activity, thereby inhibiting *S. aureus* growth [59]. In the present study, it was seen in qPCR analysis that *ompA* expression in day 1 dual-species biofilms was very high compared to *A. baumannii* monospecies biofilms (Figure 6O). Thereby, for implant infection, it was envisaged that the dominancy of *A. baumannii* in dual-species biofilms is due to its attachment to fibrinogen by high expression of *ompA*, whereas host factor binding proteins of MRSA on day 1 were downregulated, making the MRSA population low in co-culture biofilms. Fascinatingly, the population of MRSA in dual-species biofilms was raised on day 7 (Figure 4). It was suspected that MRSA competed with *A. baumannii* on day 7 for surface colonization. Genes responsible for host protein binding factors, namely *fnbA* and *fnbB* of MRSA, were increased on day 7 of dual-species biofilms (Figure 6F,G). This shows that MRSA adhesion was increased in dual-species biofilms on day 7 compared to day 1. Surprisingly, all the QS-related genes of MRSA and *A. baumannii* were downregulated in dual-species biofilms compared to monospecies biofilms developed on uncoated titanium plates. *Agr* QS regulates the production of phenol-soluble modulin (PSM), which mediates biofilm dispersion. A previous study found that *AgrAC* quorum sensing (QS) gene expression was downregulated, and host protein binding proteins were upregulated in synovial fluid compared to the microbial culture medium [60]. The same pattern of gene expression was observed when MRSA was co-cultured. The QS genes of *A. baumannii* (*abaI* and *abaR*) were also reduced when co-cultured with MRSA (Figure 6). A graphic representation of virulence gene expression in dual-species biofilms is represented in Figure 10.

Clinical translation of the Pd(II)-**E**-coated titanium plates for bone regenerative applications is possible when the material is biocompatible and exhibits osteogenic activity. Having found that the functional coating possesses superior anti-infective activity, the implants were also biocompatible with human osteoblasts (MG-63 cells). Pd(II)-**E** had no significant effect on the viability of the cells, and we believe that the ions leached from the surface of the implant coating are found to be relatively safe. Moreover, implant failure is most common among orthopedic and dental implants, with bacterial infections and a lack of osseointegration being the prime drivers of this drawback. Interestingly, in our study, the coatings promoted osteoblast differentiation and mineralization at both molecular and cellular levels. Initially, the coatings upregulated the marker genes essential for osteoblast differentiation, such as Rux2, ALP, and Col-I, and promoted matrix mineralization under osteogenic conditions (Figure 9C,D). This proves the osteoconductive nature of the implants. The formation of a mineralized layer on the implants is crucial for their integration into host bone tissue and for long-term stability. Corroborating our research findings, we conclude that Pd(II)-**E** coating on titanium implants imparted biofilm mitigating properties and osteogenic properties. These properties are crucial for its clinical success upon in vivo implantation. 

Collectively, the study confirmed that the proliferation of biofilms and extracellular matrix formation over titanium implants were high in ASF and provided a rigid biofilm architecture. The extracellular matrix of biofilms reduces the effect of antimicrobials, and in the case of polymicrobial biofilms, the treatment option creates a worrisome situation. To control biofilm infections, the present research shows an active alternative to overcome antimicrobial resistance. In the concept of “race for the surface”, when an implant is infected with a pathogen, it is difficult to achieve host-cell integration. Multiple approaches were directed at the race for the surface of host cell attachment. Our study also sheds insights into the coating of various other metallic implants with Pd(II)-**E** to enhance their biological properties. By curbing bacterial attachment without affecting bone cell attachment, the Pd(II)-**E**-coated biomaterial prefers bone cell integration to the surface of the implant. 

## 5. Conclusions

In summary, the current research showcases the successful influence of Pd(II)-**E**-fabricated titanium biomaterial for preventing orthopedic implant infections. To mimic the natural milieu of bone joints, ASF was used in the study. In vitro biofilm studies using ASF showed that Pd(II)-**E**-coated implants steadily prevented biofilm colonization. It was noticed that the aggregation of the EPS matrix was also diminished in the coated implant up to the 7th day of incubation, where reduced biomass with a lack of EPS layers was observed. Furthermore, the metal complex enhanced biocompatibility and promoted matrix mineralization, which is crucial for in vivo osseointegration. Altogether, the present study highlighted the importance of Pd(II)-**E**-coated titanium implants for biomedical applications as they assist bone cells in winning the “race for the surface” by preventing bacterial colonization. 

## Data Availability

Not Applicable.

## Supplementary Materials

The following supporting information can be downloaded at: https://www.mdpi.com/article/10.3390/antibiotics12081296/s1, Figure S1. Minimum inhibitory concentration of Pd(II)-E against dual species planktonic cells of MRSA and A. baumannii; Figure S2. Percentage biofilm inhibition of Pd(II)-E. (A) Mono-species biofilm of MRSA and A. baumannii in artificial synovial fluid. (B) Dual species biofilms of MRSA and A. baumannii in SCD and ASF. Graphs were plotted with means ± Standard deviation of triplicates. Asterisk indicated statistical difference between the control and treated groups *p* < 0.001 (***), *p* < 0.01 (**); Supplementary Figure S3. Cultivable cells of biofilm formed over titanium on days 1 (A) and 7 (B). Quantification of active biofilm cells on day 1 (C); formazan product produced by biofilms developed on day 1 (D); quantification of active biofilm cells on day 7 (E); and formazan product produced by biofilms developed on day 7 (F). SCD-soybean casein digest; ASF-Artificial synovial fluid. Graphs were plotted with means ± standard deviations of triplicates. Asterisks indicate statistical differences between the control and treated groups: *p* < 0.001 (***), *p* < 0.01 (**); Table S1. Primers of MRSA and A. baumannii; Table S2. List of primers for osteoblastic cells
