# Supplementary material for: Palladium(II) Metal Complex Fabricated Titanium Implant Mitigates Dual-Species Biofilms in Artificial Synovial Fluid
Source: Antibiotics (Basel). 2023 Aug 8;12(8):1296. doi: 10.3390/antibiotics12081296 (PMC10451766; doi:10.3390/antibiotics12081296)
Supplement: Supplementary file 1 [file antibiotics-12-01296-s001.zip › antibiotics-2502738-supplementary.pdf]

Supplementary caption

Table S1. Primers of MRSA and *A. baumannii*

Table S2. List of primers for osteoblastic cells

Supplementary Figure S1. Minimum inhibitory concentration of Pd(II)-**E** against dual-species planktonic cells of MRSA and *A. baumannii*

Supplementary Figure S2. Percentage biofilm inhibition of Pd(II)-**E**. (A) Monospecies biofilms of MRSA and *A. baumannii* in artificial synovial fluid. (B) Dual-species biofilms of MRSA and *A. baumannii* in SCD and ASF. Graphs were plotted with means ± standard deviations of triplicates. Asterisks indicate statistical differences between the control and treated groups: p<0.001(***), p<0.01(**).

Supplementary Figure S3. Cultivable cells of biofilm formed over titanium on days 1 (A) and 7 (B). Quantification of active biofilm cells on day 1 (C); formazan product produced by biofilms developed on day 1 (D); quantification of active biofilm cells on day 7 (E); formazan product produced by biofilms developed on day 7 (F). SCD-soybean casein digest; ASF-Artificial synovial fluid. Graphs were plotted with means ± standard deviations of triplicates. Asterisks indicate statistical differences between the control and treated groups: p<0.001(***), p<0.01(**).

S. No	Gene	Sequence (5'->3')	Gene function
1	*agrAC* (F)	CTGATAATCCTTATGAGGTGC	Accessory gene regulator.
	agrAC (R)	CGATGCATAGCAGTGTTC	
2	*icaA*(F)	ACACTTGCTGGCGCAGTCAA	Production of polysaccharide intercellular adhesion (PIA).
	icaA(R)	TCTGGAACCAACATCCAACA	
3	*icaD*(F)	ATGGTCAAGCCCAGACAGAG	
	icaD(R)	AGTATTTTCAATGTTTAAAGCA	
4	*crtM*(F)	ATCCAGAACCACCCGTTTTT	Synthesis of staphyloxanthin.
	crtM(R)	GCGATGAAGGTATTGGCATT	
5	*crtN*(F)	GATGAAGCTTTGACGCAACA	
	crtN(R)	TTCGCATGATACGTTTGCTC	
6	*fnbA*(F)	ATCAGCAGATGTAGCGGAAG	Adhesion of MRSA to host fibronectin.
	fnbA(R)	TTTAGTACCGCTCGTTGTCC	
7	*fnbB*(F)	AAGAAGCACCGAAAACTGTG	
	fnbB(R)	TCTCTGCAACTGCTGTAACG	
8	*sspB*(F)	CCAGCAAATTGTTGTTGTGCTAG	Survival of MRSA in serum.
	sspB(R)	AAGCCAAAGCCGATTCACACTC	
9	*csu A/B* (F)	CAGCAGCAACAGGTGGCAATA	Pili biogenesis.
	csu A/B (R)	AAGGTTTGTACGTGCAGCATCA	
10	*csuE*(F)	GCTTGGCTTTAGCAAACATGACC	Initial attachment over an abiotic surface.
	csuE(R)	ATTGCCATCAGGCCCGCTA	
11	*bfmS*(F)	ACCGCCCGTAATCCGAAC	

	bfmS(R)	TGAACTTATTCCACCGCCTTTA	Biofilm formation and surface motility.
12	*bfmR*(F)	GTTTAACCGTTTGTCGTG	Biofilm formation and surface motility.
	bfmR(R)	GTGGTTGAACTGGTTTCG	
13	*bap*(F)	TACTTCCAATCCAATGCTAGGGAGGGTACCAATGCAG	Cell-to-cell interaction.
	bap(R)	TTATCCACTTCCAATGATCAGCAACCAAACCGCTAC	
14	*pgaB*(F)	AAGAAAATGCCTGTGCCGACCA	PNAG production.
	pgaB(R)	GCGAGACCTGCAAAGGGCTGAT	
15	*ompA*(F)	CGCTTCTGCTGGTGCTGAAT	Fibronectin-binding protein, antibiotic resistance, and matrix development.
	ompA(R)	CGTGCAGTAGCGTTAGGGTA	
16	*abaR*(F)	ATGGAAAGTTGGCAAGAG	Autoinducer receptor.
	abaR(R)	CTACAAAAGCCCTAGCATTAC	
17	*abaI*(F)	ATGAATATTATTGCTGGA	Autoinducer synthase.
	abaI(R)	CTACACATCAATCAAGCA	
18	16S rRNA (F)	ACTCCTACGGGAGGCAGCAG	Internal control.
	16S rRNA (R)	ATTACCGCGGCTGCTGG	

Table S1. Primers of MRSA and *A. baumannii*

S. No	Gene	Sequence (5'->3')	Reference
1	ALP (F)	TTGTGCCAGAGAAAGAGAGAGA	Saravanan et al., 2015
	ALP (R)	GTTTCAGGGCATTTTTCAAGGT	
2	COL-I (F)	TAACCCCCTCCCCAGCCACAAA	Saravanan et al., 2015
	COL-I (R)	TTCCTCTTGGCCGTGCGTCA	
3	Runx2 (F)	CGCCTCACAAACAACCACAG	Saravanan et al., 2015
	Runx2 (R)	TCACTGTGCTGAAGAGGCTG	
4	GAPDH (F)	GAGAGACCCCACTTGCTGCCA	Saravanan et al., 2015
	GAPDH (R)	CTCACACTGCCCCTCCCTGGT	

Table S2. List of primers for osteoblastic cells

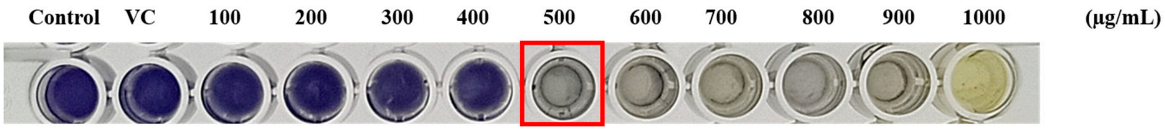

Supplementary Figure S1. Minimum inhibitory concentration of Pd(II)-**E** against dual-species planktonic cells of MRSA and *A. baumannii*.

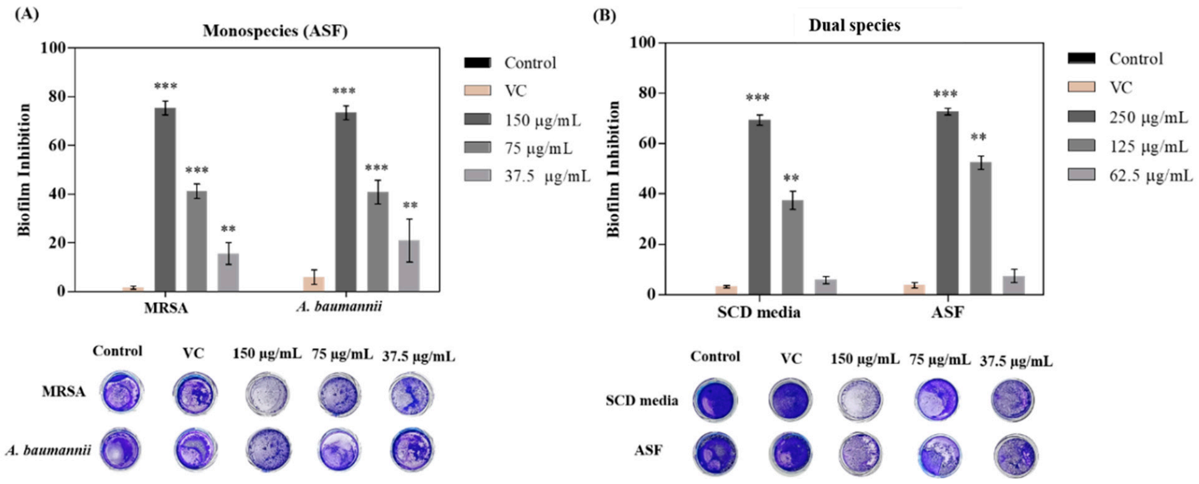

Supplementary Figure S2. Percentage biofilm inhibition of Pd(II)-**E**. (A) Monospecies biofilms of MRSA and *A. baumannii* in artificial synovial fluid. (B) Dual-species biofilms of MRSA and *A. baumannii* in SCD and ASF. Graphs were plotted with means ± standard deviations of triplicates. Asterisks indicate statistical differences between the control and treated groups: p<0.001(***), p<0.01(**).

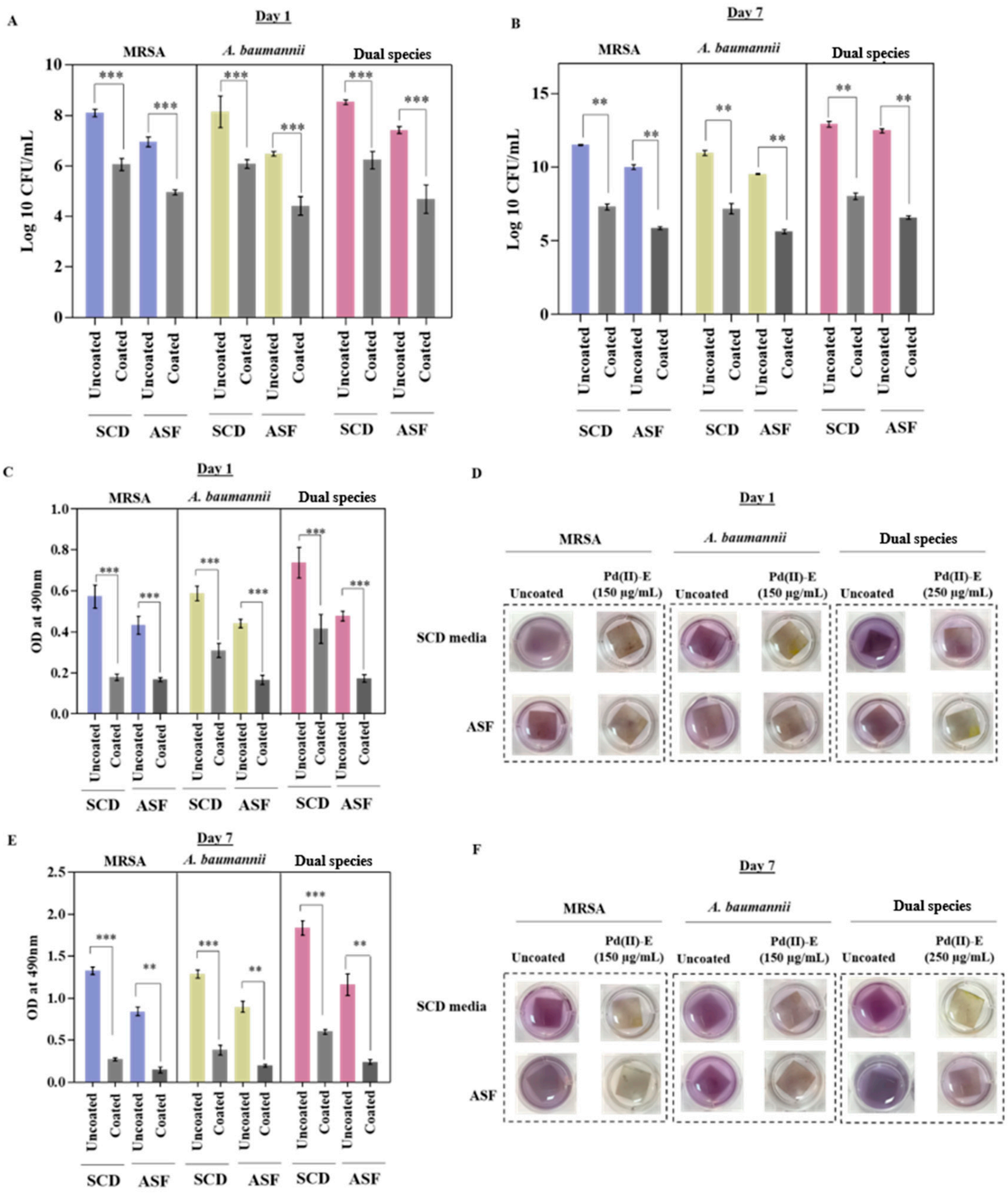

Supplementary Figure S3. Cultivable cells of biofilm formed over titanium on days 1 (A) and 7 (B). Quantification of active biofilm cells on day 1 (C); formazan product produced by biofilms developed on day 1 (D); quantification of active biofilm cells on day 7 (E); and formazan product produced by biofilms developed on day 7 (F). SCD-soybean casein digest; ASF-Artificial synovial fluid. Graphs were plotted with means ± standard deviations of triplicates. Asterisks indicate statistical differences between the control and treated groups: $p<0.001$(***), $p<0.01$(**).
